# Meta-analysis of possible role of cadherin gene methylation in evolution and prognosis of hepatocellular carcinoma with a PRISMA guideline: Erratum

**DOI:** 10.1097/MD.0000000000006990

**Published:** 2017-05-19

In the article, “Meta-analysis of possible role of cadherin gene methylation in evolution and prognosis of hepatocellular carcinoma with a PRISMA guideline”,^[1]^ which appeared in Volume 96, Issue 16 of *Medicine*, the first author's name appeared incorrectly as Chunxiao Zhu and should have appeared as Chunxia Zhu.
